# Author Correction: Accurate detection of circulating tumor DNA using nanopore consensus sequencing

**DOI:** 10.1038/s41525-023-00356-x

**Published:** 2023-06-07

**Authors:** Alessio Marcozzi, Myrthe Jager, Martin Elferink, Roy Straver, Joost H. van Ginkel, Boris Peltenburg, Li-Ting Chen, Ivo Renkens, Joyce van Kuik, Chris Terhaard, Remco de Bree, Lot A. Devriese, Stefan M. Willems, Wigard P. Kloosterman, Jeroen de Ridder

**Affiliations:** 1 Center for Molecular Medicine and Oncode Institute, University Medical Center Utrecht, Utrecht University, Utrecht, CX The Netherlands; 2 Cyclomics, Utrecht, CG The Netherlands; 3 Department of Genetics, University Medical Center Utrecht, Utrecht University, Utrecht, CX The Netherlands; 4 Department of pathology, University Medical Center Utrecht, Utrecht University, Utrecht, CX The Netherlands; 5 Department of Oral and Maxillofacial Surgery, University Medical Center Utrecht, Utrecht University, Utrecht, CX The Netherlands; 6 Department of Radiotherapy, UMC Utrecht Cancer Center, University Medical Center Utrecht, Utrecht, The Netherlands; 7 Department of Head and Neck Surgical Oncology, UMC Utrecht Cancer Center, University Medical Center Utrecht, Utrecht, The Netherlands; 8 Department of Medical Oncology, UMC Utrecht Cancer Center, University Medical Center Utrecht, Utrecht, The Netherlands; 9 Center for Molecular Medicine, University Medical Center Utrecht, Utrecht University, Utrecht, CX The Netherlands; 10 Present Address: Department of Pathology and Medical Biology, University Medical Center Groningen, Rijksuniversiteit Groningen, Groningen, GZ The Netherlands

**Keywords:** Diagnostic markers, DNA sequencing

Correction to: *npj Genomic Medicine* 10.1038/s41525-021-00272-y, published online 09 December 2021.

The Data Availability statement in the original version of the paper reads: “The sequencing datasets generated during the current study are available upon request at EGA, under accession number EGAS00001003759”. However, as this data upload was not successful, the authors reuploaded the data under a different accession number and have amended the Data Availability statement to read “The sequencing datasets generated during the current study are available upon request at EGA, under accession number EGAS00001007090”. The original article has been corrected.

