# Supplementary material for: Accurate detection of circulating tumor DNA using nanopore consensus sequencing
Source: NPJ Genom Med. 2021 Dec 9;6:106. doi: 10.1038/s41525-021-00272-y (PMC8660781; doi:10.1038/s41525-021-00272-y)
Supplement: Supplementary file 1 — Supplementary Information [file 41525_2021_272_MOESM1_ESM.pdf]

Supplementary Material

Accurate detection of circulating tumor DNA using nanopore consensus sequencing

Alessio Marcozzi[1,2,#], Myrthe Jager[1,#], Martin Elferink[3], Roy Straver[1], Joost H. van Ginkel[4,5], Boris Peltenburg[6,7], Li-Ting Chen[1], Ivo Renkens[1], Joyce van Kuik[4], Chris Terhaard[6], Remco de Bree[7], Lot A. Devriese[8], Stefan M. Willems[4,9], Wigard Kloosterman[2,10,*] and Jeroen de Ridder[1,2,*].

[1] Center for Molecular Medicine and Oncode Institute, University Medical Center Utrecht, Utrecht University, Heidelberglaan 100, 3584 CX Utrecht, The Netherlands.

[2] Cyclomics, Universiteitsweg 100, 3584 CG Utrecht, The Netherlands.

[3] Department of Genetics, University Medical Center Utrecht, Utrecht University, Heidelberglaan 100, 3584 CX Utrecht, The Netherlands.

[4] Department of pathology, University Medical Center Utrecht, Utrecht University, Heidelberglaan 100, 3584 CX Utrecht, The Netherlands.

[5] Department of Oral and Maxillofacial Surgery, University Medical Center Utrecht, Utrecht University, Heidelberglaan 100, 3584 CX Utrecht, The Netherlands.

[6] Department of Radiotherapy, UMC Utrecht Cancer Center, University Medical Center Utrecht, Utrecht, The Netherlands. [7] Department of Head and Neck Surgical Oncology, UMC Utrecht Cancer Center, University Medical Center Utrecht, Utrecht, The Netherlands.

[8] Department of Medical Oncology, UMC Utrecht Cancer Center, University Medical Center Utrecht, Utrecht, The Netherlands.

[9] Current address: Department of Pathology and Medical Biology, University Medical Center Groningen, Rijksuniversiteit Groningen, Hanzeplein 1, 9713 GZ Groningen, The Netherlands.

[10] Center for Molecular Medicine, University Medical Center Utrecht, Utrecht University, Heidelberglaan 100, 3584 CX Utrecht, The Netherlands.

[#] These authors contributed equally.

* Corresponding authors: wigard@cyclomics.com, J.deridder-4@umcutrecht.nl.

Supplementary Figures

Supplementary Figure 1	Detailed CyclomicsSeq protocol.
Supplementary Figure 2	Approach to design the three backbones used
Supplementary Figure 3	Reads distribution grouped by insert size
Supplementary Figure 4	Reproducibility of CyclomicsSeq in six technical replicates
Supplementary Figure 5	Consensus calling lowers the error rate in the backbone
Supplementary Figure 6	The effect of forward/reverse correction is consistent between runs
Supplementary Figure 7	Mean single-nucleotide false positive rate across BB22 and BB25
Supplementary Figure8	Consensus calling lowers the error rate in Flongle flow cells
Supplementary Figure 9	Consensus calling lowers the error rate in R10 flow cells
Supplementary Figure 10	Detecting mutations in a single synthetic TP53 exon in pJET

Supplementary Tables

Supplementary Table 1	Sequences of backbones and inserts
Supplementary Table 2	Sample information
Supplementary Table 3	Sequencing information

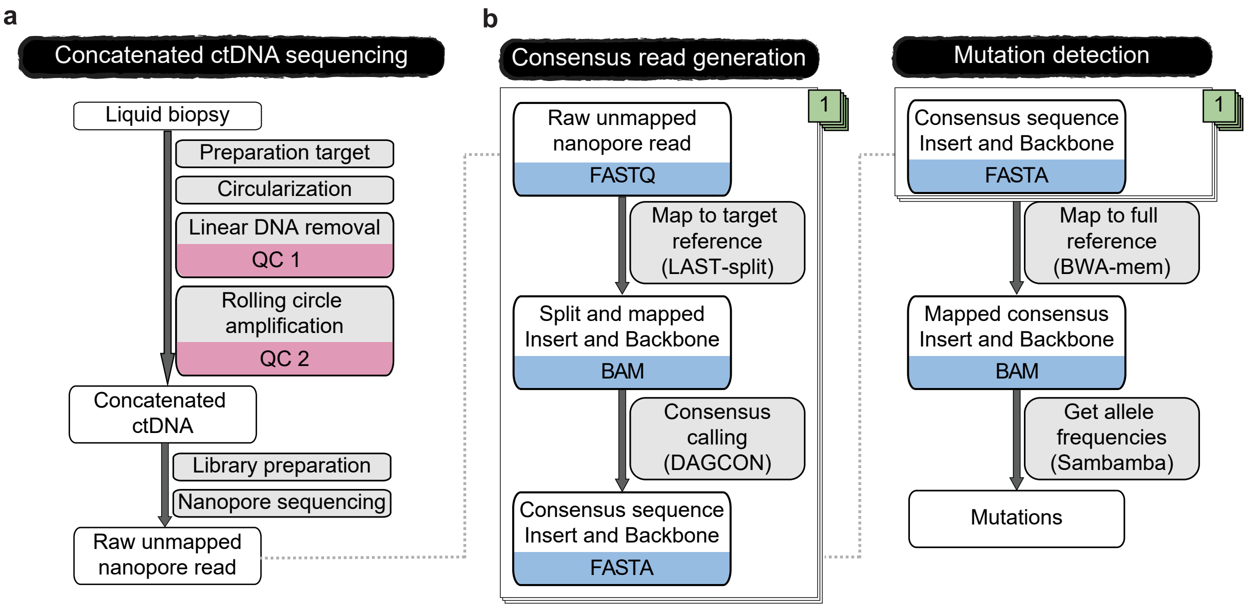

Supplementary Figure 1. Detailed CyclomicsSeq protocol. a Schematic overview of the wet-lab steps of CyclomicsSeq. QC = quality control. **b** Schematic overview of the dry-lab steps of CyclomicsSeq.

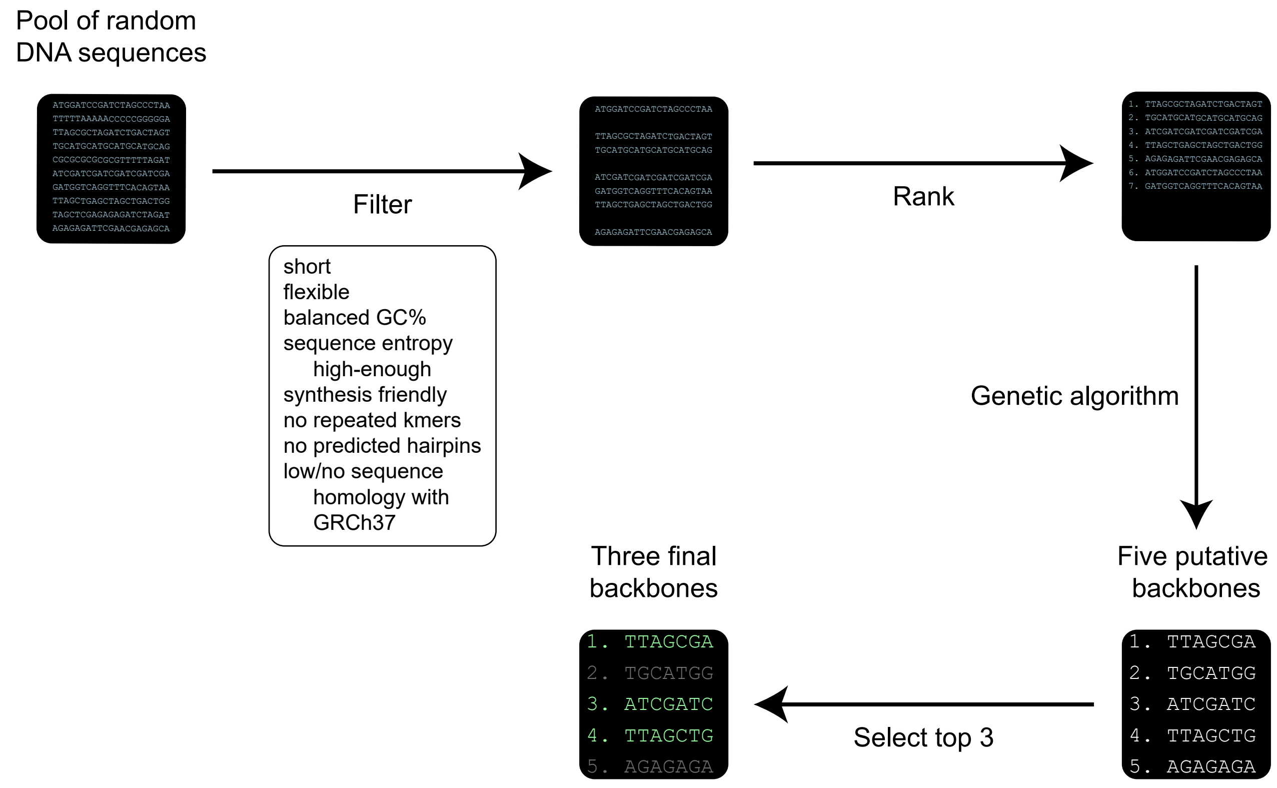

Supplementary Figure 2. Approach to design the three backbones used: BB22, BB24 and BB25.

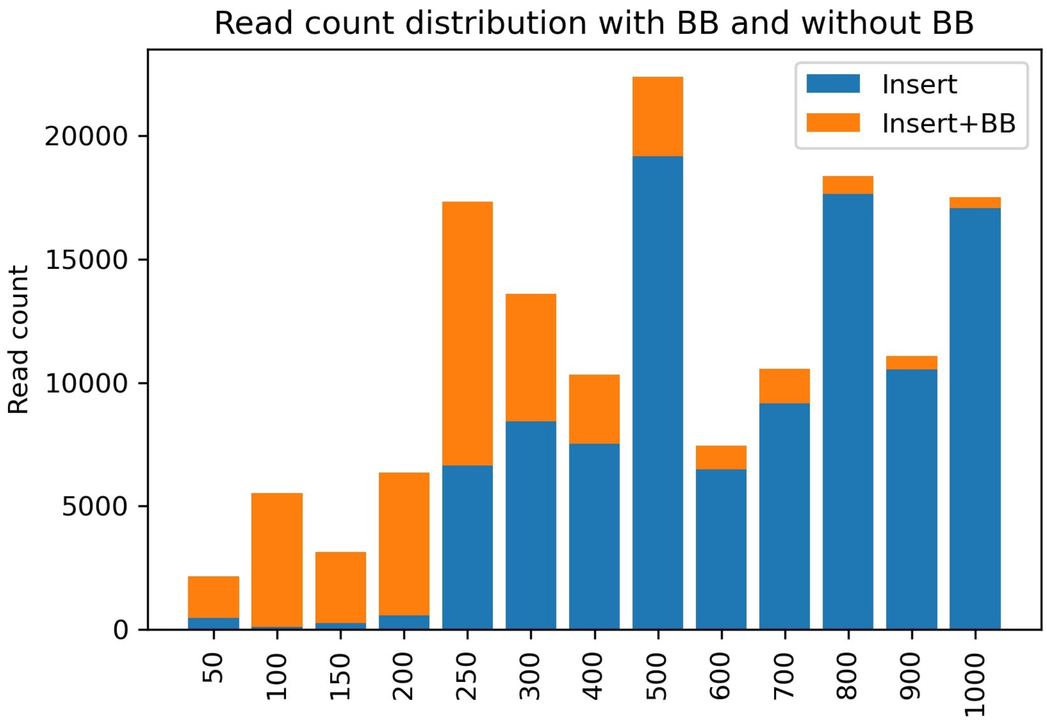

Supplementary Figure 3. Reads distribution grouped by insert size. Concatemeric reads were grouped by insert size (X axis) and colored based on the presence (orange) or absence (blue) of a backbone (BB) in the repeated unit.

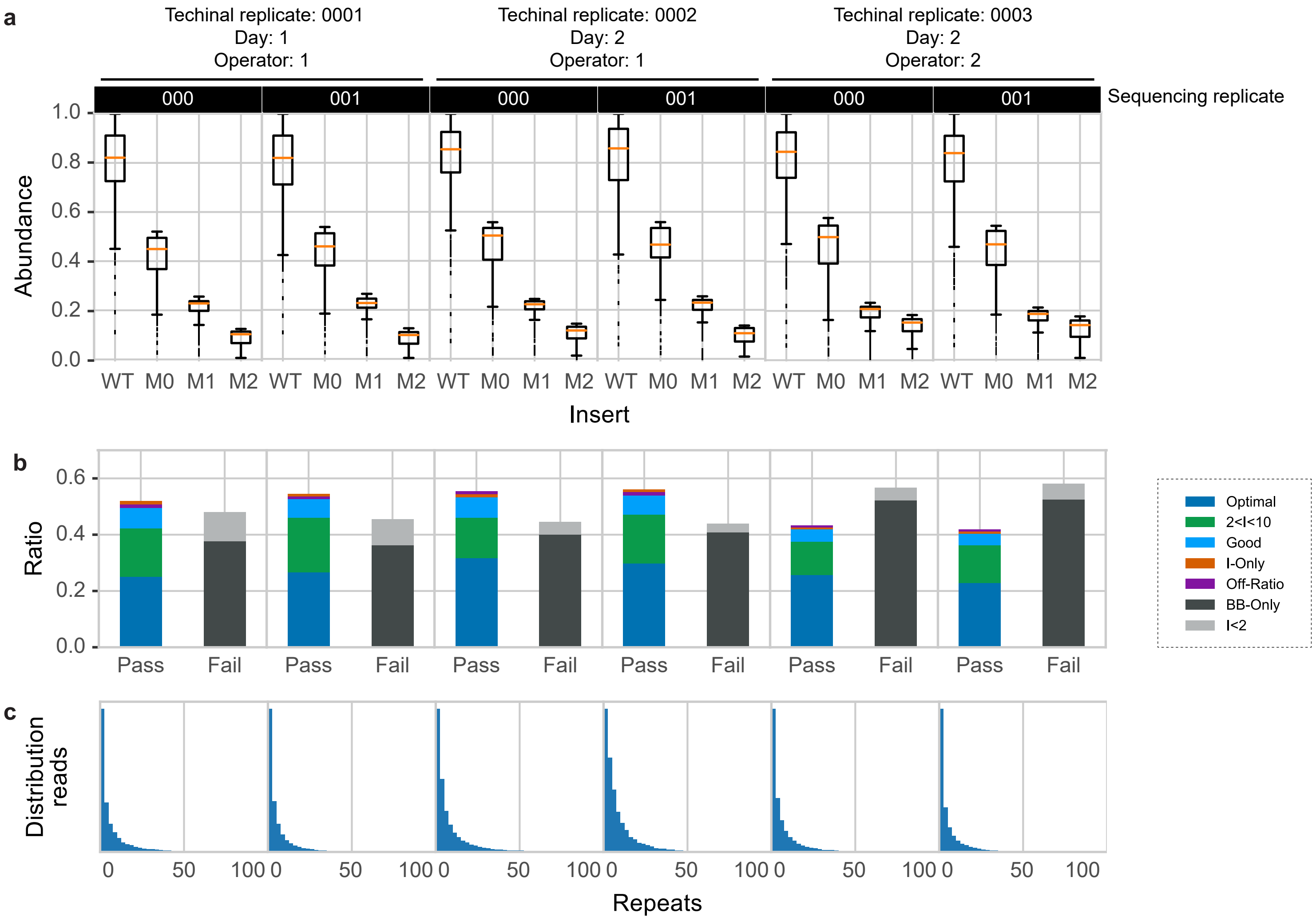

Supplementary Figure 4. Reproducibility of CyclomicsSeq in six technical replicates. a Box plots (center line = median; box limits = 25th and 75th percentiles; whiskers = 1.5x interquartile range; data points = outliers) depicting abundance of four inserts observed across the six technical replicates. WT = Wild type. M0 = Mutant 0, M1 = Mutant 1, M2 = Mutant 2. **b** Ratio of sequencing data grouped by read type for CyclomicsSeq reads. Colors, noted in the legend, represent the different categories a read can belong to (see Figure 1 for a description). **c** Number of repeats versus the number of reads. 6 Flongle runs were used for these analyses. Day of processing and operator are indicated above the graphs. 000 and 001 are sequencing replicates of the same technical replicate.

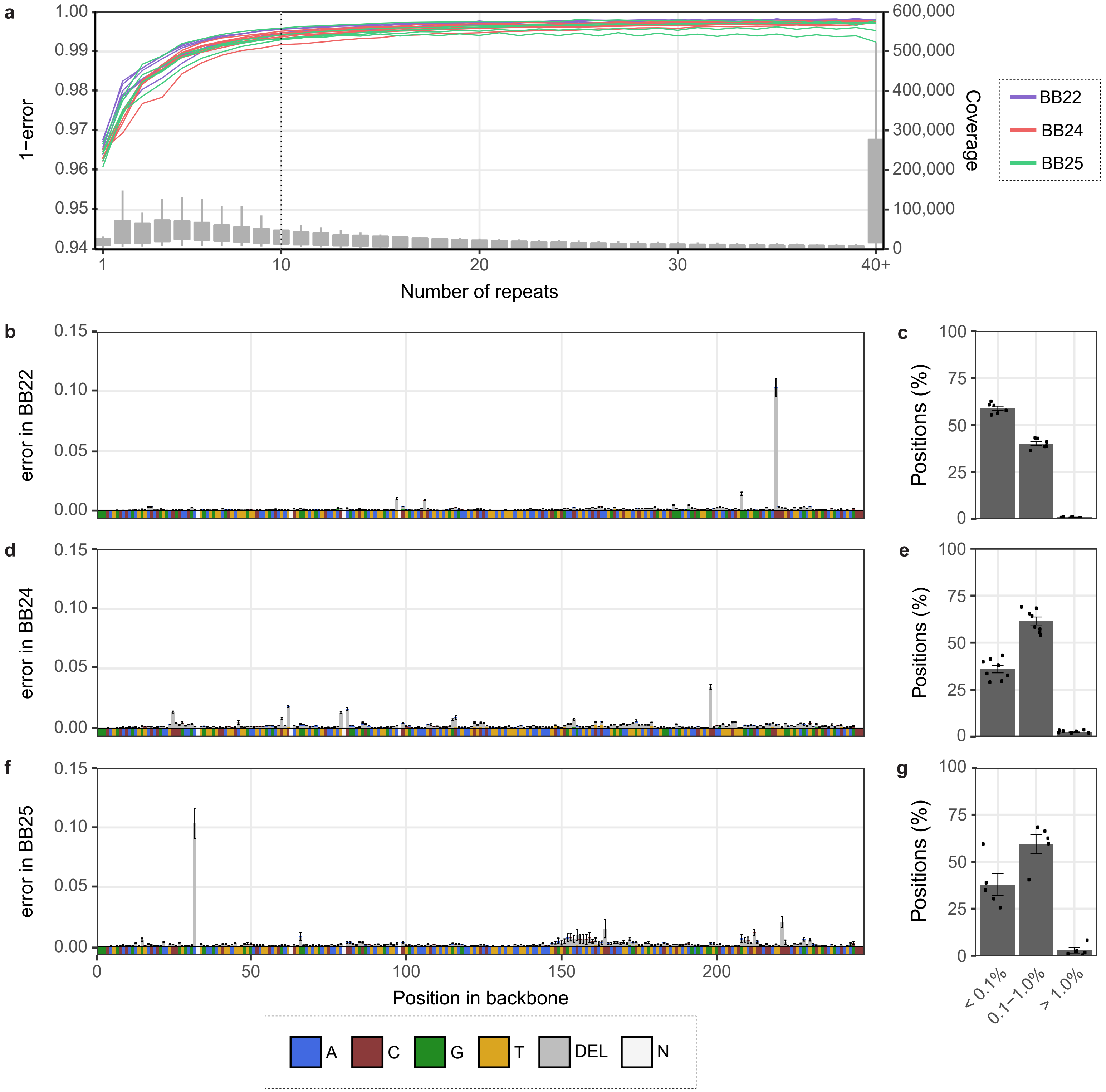

Supplementary Figure 5. Consensus calling lowers the error rate in the backbone. a 1 - Error rate (FP + deletions) in backbones (BB22, BB24, and BB25) per number of repeats. The dashed line indicates 10 repeats. Colours represent backbone type. Mean error rate across BB22 (**b**), BB24 (**d**) and BB25 (**f**) in reads with at least 10 repeats. Reference sequence is depicted below the x-axis. Colours represent base type. N = any. Percentage of positions in BB22 (**c**), BB24 (**e**) and BB25 (**g**) with indicated error percentage. Data points represent individual sequencing runs. 6 BB22, 8 BB24, and 5 BB25 runs were used for the calculations. Error bars indicate the standard deviation (sd).

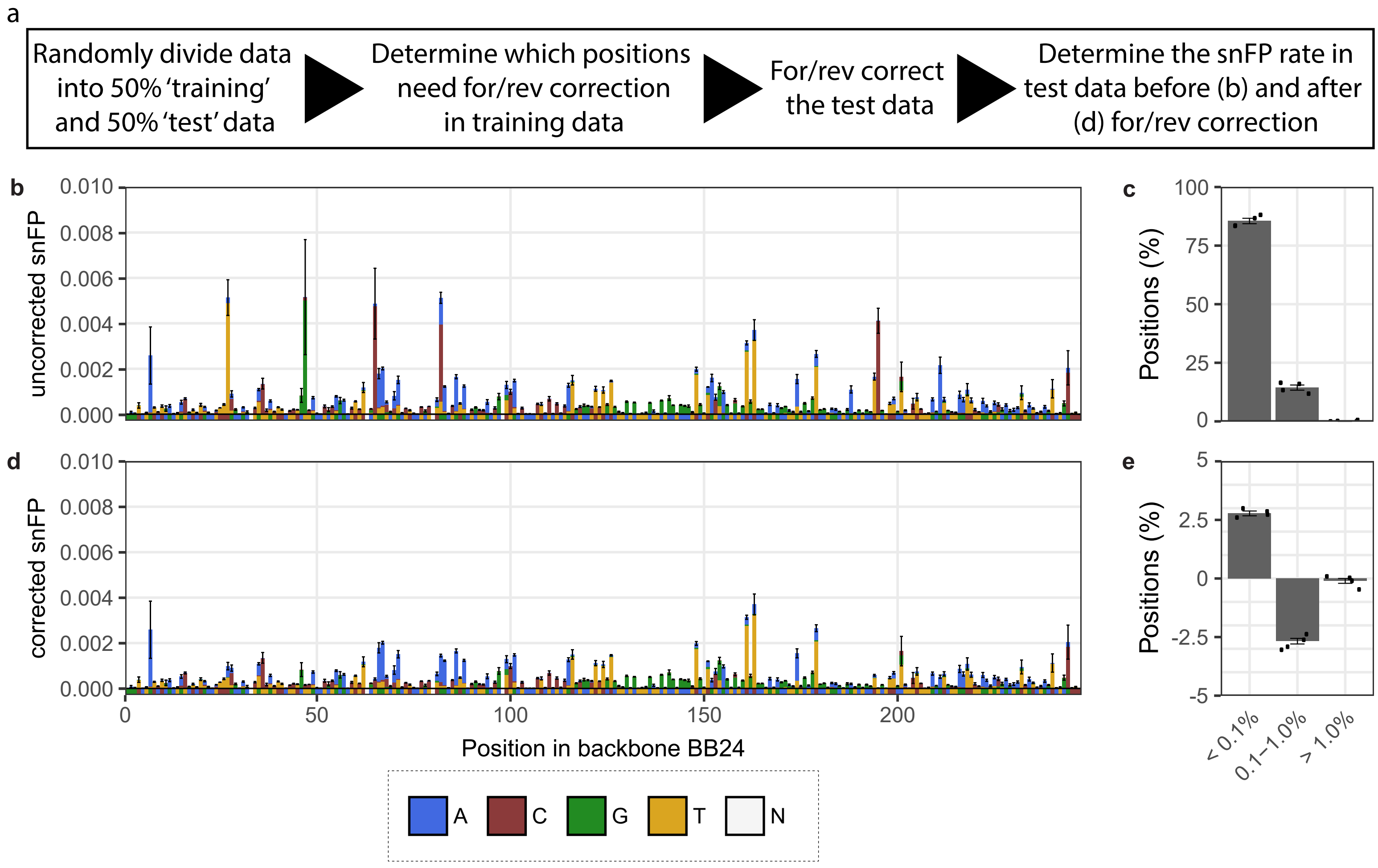

Supplementary Figure 6. The effect of forward/reverse correction is consistent between runs. a Approach to determine whether the results of the forward/reverse correction are reproducible. The samples were divided into a 50% 'training' and a 50% 'test' set. Next, the bases that require forward/reverse correction were identified in the 'training' data. The forward/reverse correction was subsequently applied to the 'test data'. Finally, the snFP rate before and after forward/reverse correction was compared in the 'test data'. **b** Mean snFP rate across BB24 in the 'test data' in reads with at least 10 repeats prior to and **d** after forward/reverse correction. Reference sequence is depicted below the x-axis. Colours represent base type. N = any. **c** Percentage of positions in BB24 with indicated snFP percentage in the 'test data' prior to forward/reverse correction and **e** the difference after forward/reverse correction. Data points represent individual sequencing runs. 8 BB24 runs were used for the calculations. Error bars indicate the standard deviation (sd). snFP = single nucleotide false positive.

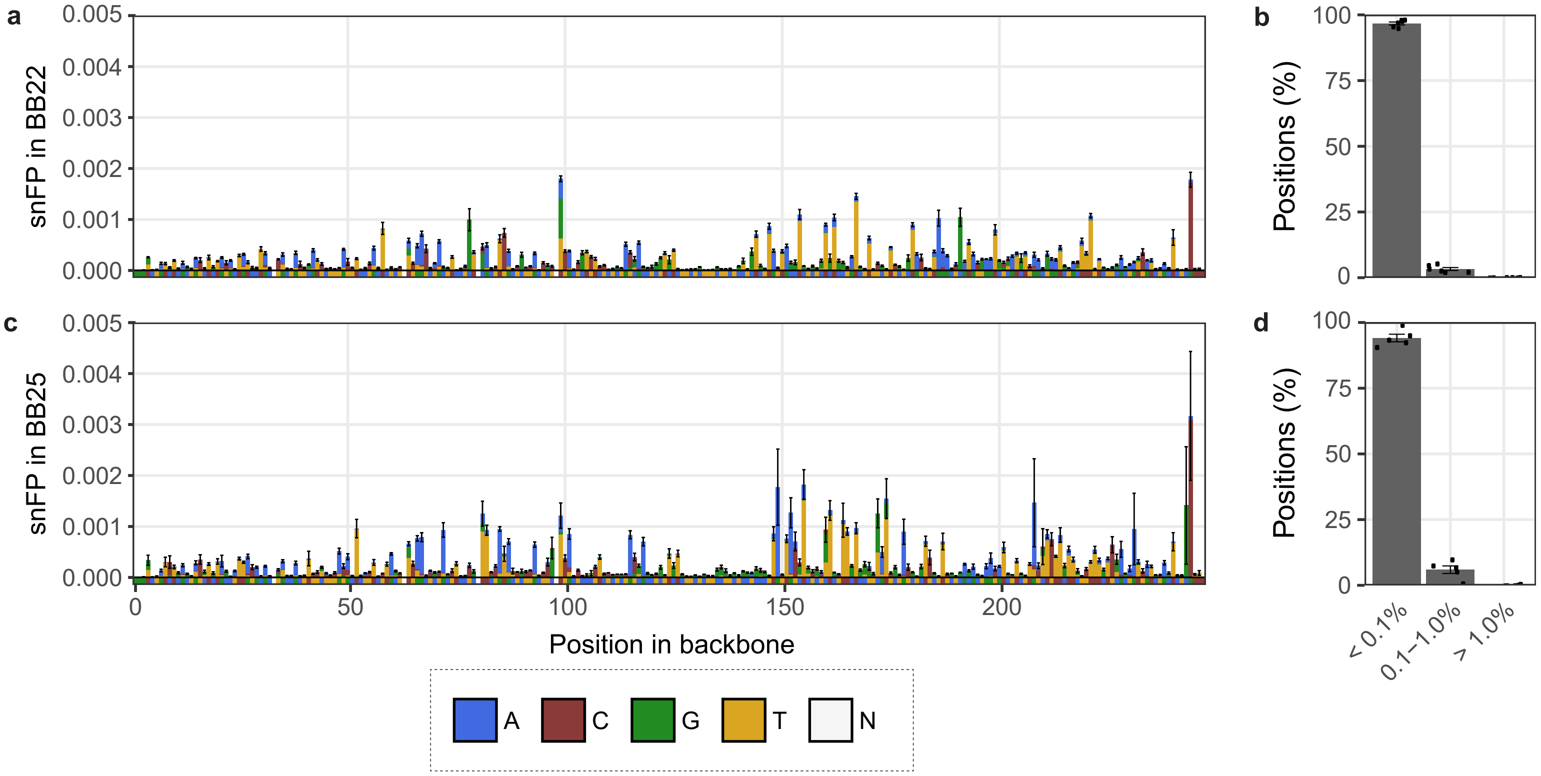

Supplementary Figure 7. Mean single-nucleotide false positive rate across BB22 (**a**) and BB25 (**c**) in reads with at least 10 repeats. Reference sequence is depicted below the x-axis. Colours represent base type. N = any. Percentage of positions in BB22 (**b**) and BB25 (**d**) with indicated snFP percentage. Data points represent individual sequencing runs. 6 BB22 and 5 BB25 runs were used for the calculations. Error bars indicate the standard deviation (sd).

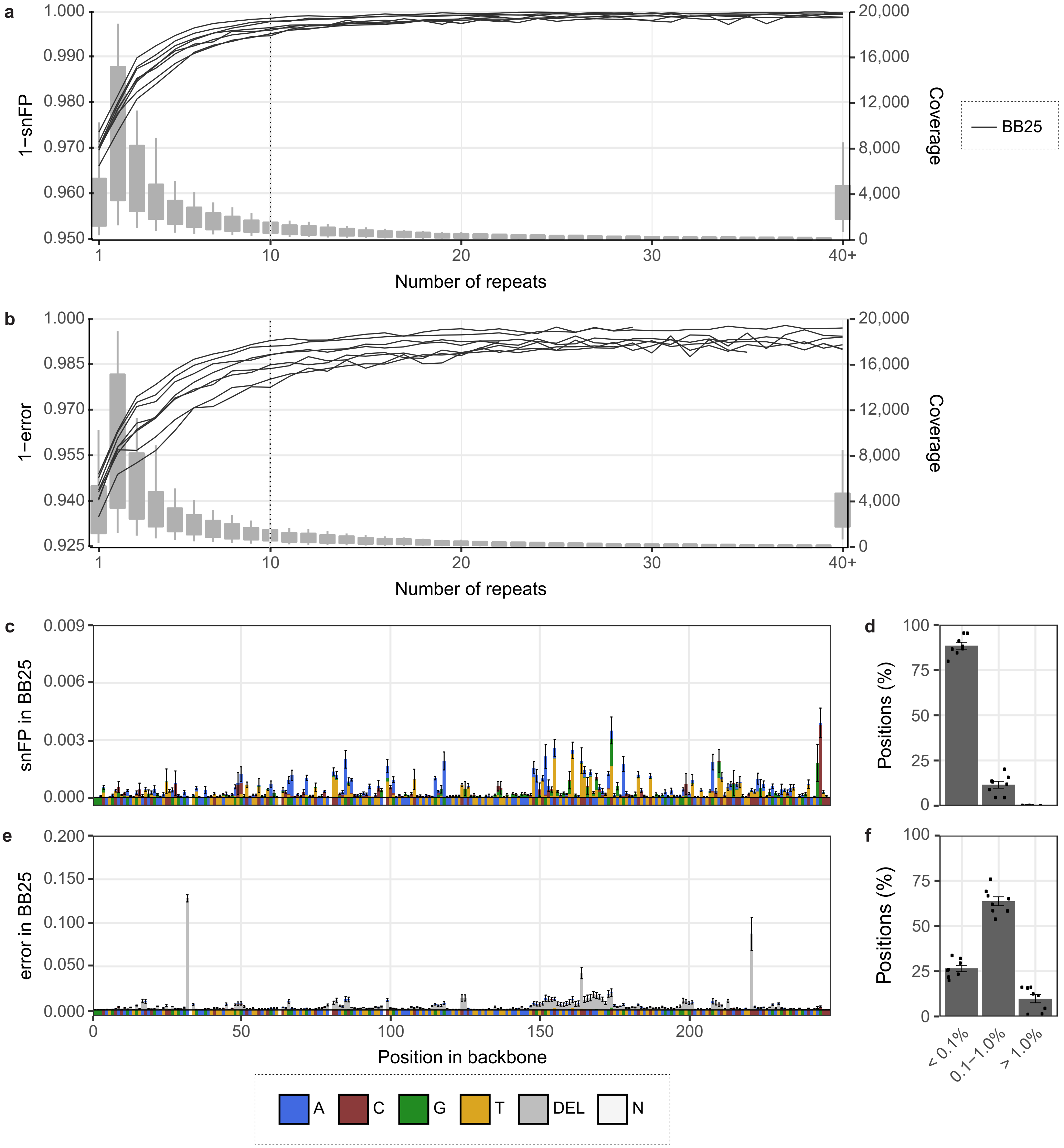

Supplementary Figure 8. Consensus calling lowers the number of sequencing errors in the backbone sequenced with Flongle. a 1 - single nucleotide false positive (snFP) and **b** 1- error (snFP + deletion) in backbone BB25 per number of repeats. The dashed line indicates 10 repeats. **c** Mean snFP and e error rate across BB25 in reads with at least 10 repeats. Reference sequence is depicted below the x-axis. Colours represent base type. N = any. **d** Percentage of positions in BB25 with indicated snFP and **f** error percentage. Data points represent individual sequencing runs. 8 BB25 runs were used for the calculations. Error bars indicate the standard deviation (sd).

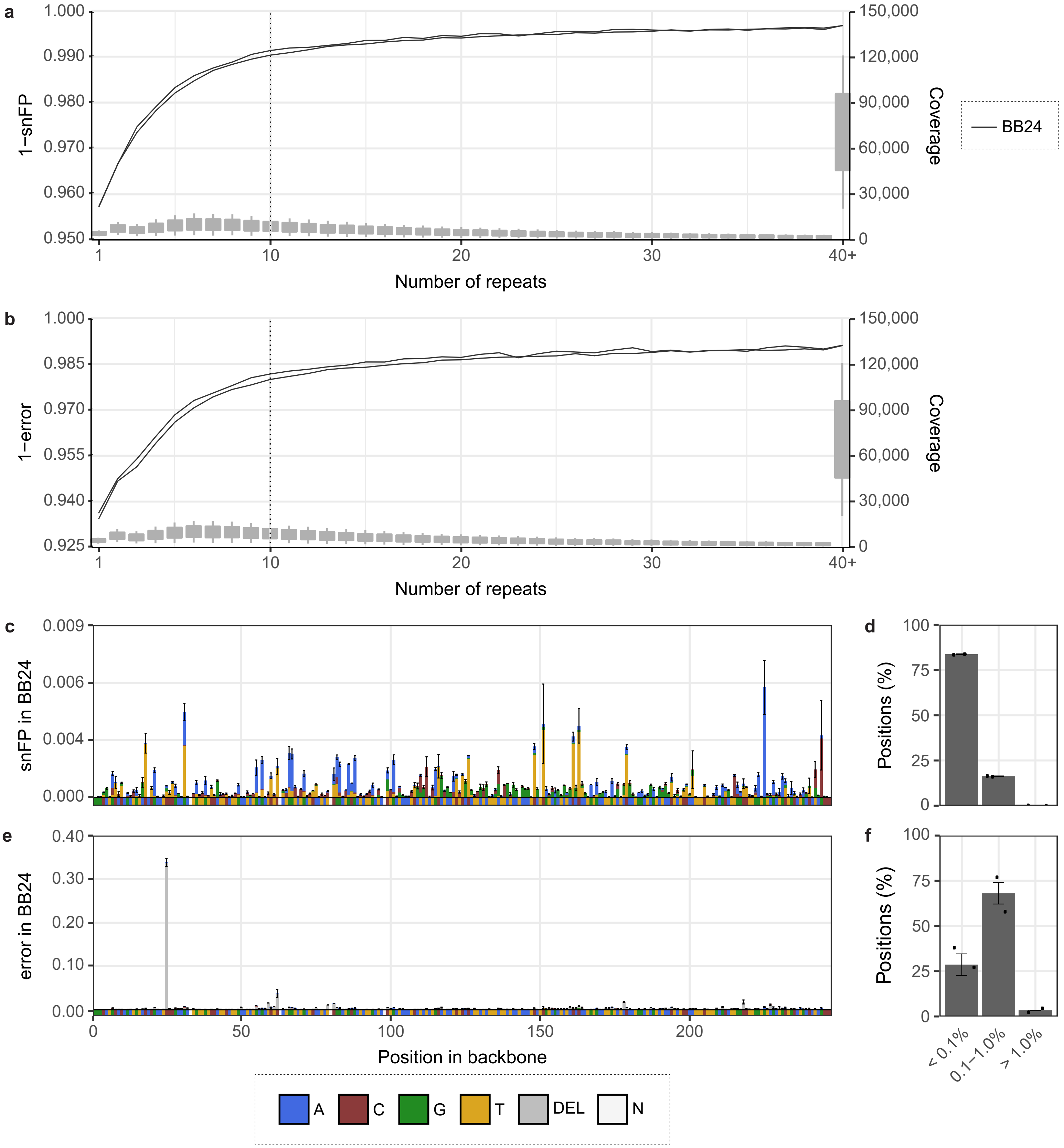

Supplementary Figure 9. Consensus calling lowers the number of sequencing errors in the backbone sequenced with R10 flow cells. **a** 1 - single nucleotide false positive (snFP) and **b** 1- error (snFP + deletion) in backbone BB24 per number of repeats. The dashed line indicates 10 repeats. **c** Mean snFP and **e** error rate across BB24 in reads with at least 10 repeats. Reference sequence is depicted below the x-axis. Colours represent base type. N = any. **d** Percentage of positions in BB24 with indicated snFP and **f** error percentage. Data points represent individual sequencing runs. 2 BB24 runs were used for the calculations. Error bars indicate the standard deviation (sd).

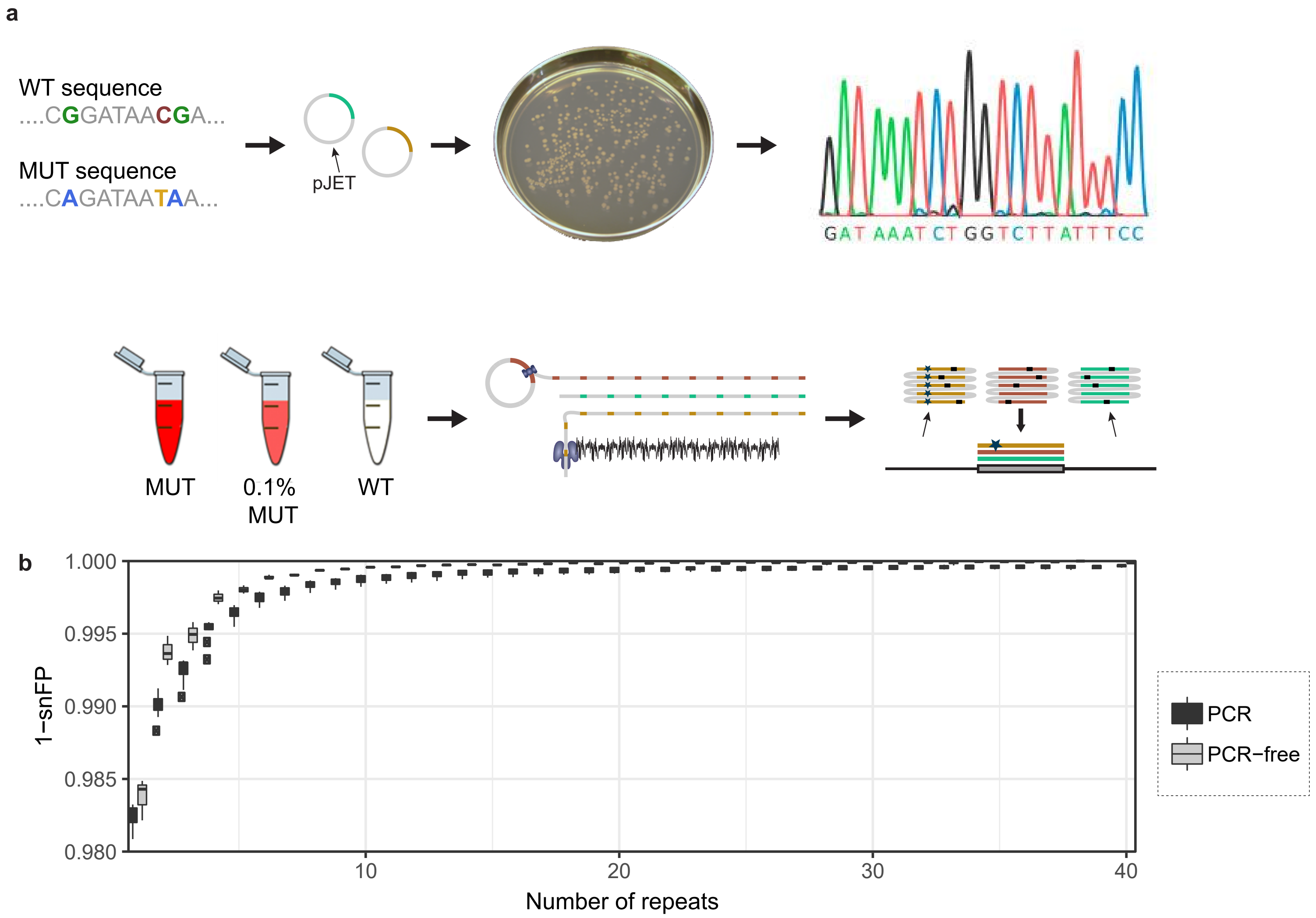

Supplementary Figure 10. Detecting mutations in a single synthetic *TP53* exon in pJET using CyclomicsSeq. a Experimental setup of the experiment. A synthetic WT sequence and MUT sequence (with three common COSMIC mutations in HNSCC) were cloned into pJET. Both sequences were amplified *in vivo* (PCR-free). Sanger sequencing was used to confirm the correctness of the WT and MUT sequence. CyclomicsSeq was performed on 100% WT, 100% MUT, and WT supplemented with 0.1% MUT. **b** Box plots (center line = median; box limits = 25th and 75th percentiles; whiskers = 1.5x interquartile range; data points = outliers) depicting 1 - single nucleotide false positive (snFP) rate in the insert (17:7577010-7577150 in GRCh37) per number of repeats for 8 PCR and 3 PCR-free inserts.

Name	Genomic coordinates	Sequence
BB22	NA	GGGCATGCACAGATGTACACGTGACGCAACGANTGATGTTAGCTATTTGTTCAATGACATATNCTGGTATGATCAATACNAGATCTGATAT TGATATNCTGATACTCATATATGTAGAATATCACATTATATTTATTATAATACATCGTCGAACATATACACAATGCATCTTATCTATACGTAT CGGGATAGCGTTGGCATAGCACTGGATGGCATGACCCTCATTAGATGCTGCATGACATAGCCC
BB24	NA	GGGCATGCACAGATGTACACGAATCCCGAAGANTGTTGTCCATTCATTGAATATGAGATCTCNATGGTATGATCAATATNCGGATGCGAT ATTGATANCTGATAAATCATATATGCATAATCTCACATTATATTTATTATAATAAATCATCGTAGATATACACAATGTGAATTGTATACAATG GATAGTATAACTATCCAATTTCTTTGAGCATTGGCCTTGGTGTAGATGCTGCATGACATAGCCC
BB25	NA	GGGCATGCACAGATGTACACGAATCCGTGAGANTGAAGATCTTATTTGTGACATTCATCGATNCTGGATATGATCAATANCCATGCGATAT TGATTANCTGATAAATCATATATGTAGAATATCACATTATATTAATTATAATAAATCGTCGTACATATACATCCACAATTAGCTATGTATACT ATCTATAGAGATGGTGCATCATCGTACTCCACCATTCCCACTAGATGCTGCATGACATAGCCC
pJET	NA	See https://assets.thermofisher.com/TFS-Assets/LSG/brochures/pJET1.2-plasmid-sequence.txt
TP53 amplicon5	17:7573954-7574071	TCCTTCCCAGCCTGGGCATCCTTGAGTTCCAAGGCCTCATTCAGCTCTCGGAACATCTCGAAGCGCTCACGCCCACGGATCTGCAGCAACA GAGGAGGGGGAGAAGTAAGTATATACA
TP53 amplicon9	17:7576810-7576955	ACTGGAAACTTTCCACTTGATAAGAGGTCCCAAGACTTAGTACCTGAAGGGTGAAATATTCTCCATCCAGTGGTTTCTTCTTTGGCTGGGGA GAGGAGCTGGTGTTGTTGGGCAGTGCTAGGAAAGAGGCAAGGAAAGGTGATAAA
TP53 amplicon12	17:7577010-7577150	CTTGCTTACCTCGCTTAGTGCTCCCTGGGGGCAGCTCGTGGTGAGGCTCCCCTTTCTTGCGGAGATTCTCTTCCTCTGTGCGCCGGTCTCTCC CAGGACAGGCACAAACACGCACCTCAAAGCTGTTCCGTCCCAGTAGAT
TP53 amplicon19	17:7578343-7578452	TGTCGTCTCTCCAGCCCCAGCTGCTCACCATCGCTATCTGAGCAGCGCTCATGGTGGGGGCAGCGCCTCACAACCTCCGTCATGTGCTGTG ACTGCTTGTAGATGGCCAT
TP53 amplicon20	17:7578409-7578545	CTCACAACCTCCGTCATGTGCTGTGACTGCTTGTAGATGGCCATGGCGCGGACGCGGGTGCCGGGCGGGGGTGTGGAATCAACCCACAG CTGCACAGGGCAGGTCTTGGCCAGTTGGCAAAACATCTTGTTGAGGGC
TP53 amplicon21	17:7578476-7578592	GGGGTGTGGAATCAACCCACAGCTGCACAGGGCAGGTCTTGGCCAGTTGGCAAAACATCTTGTTGAGGGCAGGGGAGTACTGTAGGAAG AGGAAGGAGACAGAGTTGAAAGTCAGGG
TP53 ampliconS0	17:7579205-7579355	GAAGCCAAAGGGTGAAGAGGAATCCCAAAGTTCCAAACAAAAGAAATGCAGGGGGATACGGCCAGGCATTGAAGTCTCATGGAAGCCA GCCCCTCAGGGCAACTGACCGTGCAAGTCACAGACTTGGCTGTCCCAGAATGCAAGAAGCCCA

Supplementary Table 1. Sequences of backbones and inserts

Sample ID	Sample name	Sample type	Sample source	Mutation	Time point
CY_SS_PC_HC_0001_001_000	Control A	Healthy control	Plasma cfDNA	NA	NA
CY_SM_PC_HC_0002_001_000	Control B	Healthy control	Plasma cfDNA	NA	NA
CY_SM_PC_HC_0004_001_000	Control C	Healthy control	Plasma cfDNA	NA	NA
CY_SM_PC_HC_0004_002_000	Control C	Healthy control	Plasma cfDNA	NA	NA
CY_SM_PC_HC_0004_003_000	Control C	Healthy control	Plasma cfDNA	NA	NA
CY_SM_PC_HC_0004_004_000	Control C	Healthy control	Plasma cfDNA	NA	NA
CY_BB25_19WT_0001_000	Control D	Healthy control	Plasma cfDNA	NA	NA
CY_SS_PC_HC_0005_002_000	Control E	Healthy control	Plasma cfDNA	NA	NA
CY_SS_PC_HN_0001_001_000	Patient A 0B	HNSCC	Plasma cfDNA	17:7577121 G>A	0
CY_SS_PC_HN_0001_002_000	Patient A 1B	HNSCC	Plasma cfDNA	17:7577121 G>A	1
CY_SS_PC_HN_0001_005_000	Patient A 1B	HNSCC	Plasma cfDNA	17:7577121 G>A	1
CY_SS_PC_HN_0001_003_000	Patient A 2B	HNSCC	Plasma cfDNA	17:7577121 G>A	2
CY_SS_SC_HN_0001_006_000	Patient A 2S	HNSCC	Saliva cfDNA	17:7577121 G>A	2
CY_SS_PC_HN_0001_004_000	Patient A 3B	HNSCC	Plasma cfDNA	17:7577121 G>A	4
CY_SM_PC_HN_0002_001_000	Patient B 0B	HNSCC	Plasma cfDNA	17:7577095-7577123 Deletion	0
CY_SM_PC_HN_0002_002_001	Patient B 0B	HNSCC	Plasma cfDNA	17:7577095-7577123 Deletion	0
CY_SM_PC_HN_0002_003_000	Patient B 1B	HNSCC	Plasma cfDNA	17:7577095-7577123 Deletion	1
CY_SS_PC_HN_0003_001_000	Patient C 0B	HNSCC	Plasma cfDNA	17:7578403 C>T	0
CY_SS_PC_HN_0003_006_000	Patient C 19B	HNSCC	Plasma cfDNA	17:7578403 C>T	19
CY_SS_PC_HN_0003_002_000	Patient C 1B	HNSCC	Plasma cfDNA	17:7578403 C>T	1
CY_SS_PC_HN_0003_003_000	Patient C 2B	HNSCC	Plasma cfDNA	17:7578403 C>T	2
CY_SS_PC_HN_0003_004_000	Patient C 3B	HNSCC	Plasma cfDNA	17:7578403 C>T	3
CY_SS_PC_HN_0003_005_000	Patient C 4B	HNSCC	Plasma cfDNA	17:7578403 C>T	4
CY_PJET_12MU_0001_000	MUT	Synthetic control	Synthetic	17:7577094 G>A ; 17:7577120 C>T ; 17:7577121 G>A	NA
CY_LOT1_QC_0001_000	REP mix 1	Synthetic control	Synthetic	Multiple mixed	NA
CY_LOT1_QC_0001_001	REP mix 1	Synthetic control	Synthetic	Multiple mixed	NA
CY_LOT1_QC_0002_000	REP mix 2	Synthetic control	Synthetic	Multiple mixed	NA
CY_LOT1_QC_0002_001	REP mix 2	Synthetic control	Synthetic	Multiple mixed	NA
CY_LOT1_QC_0003_000	REP mix 3	Synthetic control	Synthetic	Multiple mixed	NA
CY_LOT1_QC_0003_001	REP mix 3	Synthetic control	Synthetic	Multiple mixed	NA
CY_PJET_12WT_0001_000	WT	Synthetic control	Synthetic	NA	NA
CY_PJET_RATI_0001_000	WT/MUT mix	Synthetic control	Synthetic	17:7577094 G>A ; 17:7577120 C>T ; 17:7577121 G>A	NA

NA = Not applicable; WT = Wild type; MUT = Mutant; REP = reproducibility; HNSCC = head and neck squamous cell carcinoma; cfDNA = cell free DNA

Supplementary Table 2. Sample information

Sample ID	Backbone	Insert	Flow cell	Reads
CY_SS_PC_HC_0001_001_000	BB24	*TP53* amplicon 12	R9	1459482
CY_SM_PC_HC_0002_001_000	BB24	*TP53* amplicon 5,9	R9	1783724
CY_SM_PC_HC_0004_001_000	BB25	*TP53* amplicon 9,19,20,21	R9	3681526
CY_SM_PC_HC_0004_002_000	BB25	*TP53* amplicon 9,19,20,21	Flongle	73591
CY_SM_PC_HC_0004_003_000	BB25	CleanPlex *TP53* Panel*	R9	2450755
CY_SM_PC_HC_0004_004_000	BB25	CleanPlex *TP53* Panel*	Flongle	173694
CY_BB25_19WT_0001_000	BB25	*TP53* amplicon 19	R9	11854864
CY_SS_PC_HC_0005_002_000	BB25	*TP53* amplicon 12	R9	5345433
CY_SS_PC_HN_0001_001_000	BB24	*TP53* amplicon 12	R9	1340417
CY_SS_PC_HN_0001_002_000	BB24	*TP53* amplicon 12	R9	1343902
CY_SS_PC_HN_0001_005_000	BB24	*TP53* amplicon 12	R10	585206
CY_SS_PC_HN_0001_003_000	BB24	*TP53* amplicon 12	R9	1089610
CY_SS_SC_HN_0001_006_000	BB24	*TP53* amplicon 12	R9	1583579
CY_SS_PC_HN_0001_004_000	BB24	*TP53* amplicon 12	R9	860412
CY_SM_PC_HN_0002_001_000	BB24	*TP53* amplicon 5,9,12	R9	1019983
CY_SM_PC_HN_0002_002_001	BB24	*TP53* amplicon 5,9,12	R10	108000
CY_SM_PC_HN_0002_003_000	BB25	*TP53* amplicon 9	R9	2453166
CY_SS_PC_HN_0003_001_000	BB22	*TP53* amplicon 19	R9	3776045
CY_SS_PC_HN_0003_006_000	BB22	*TP53* amplicon 19	R9	4680602
CY_SS_PC_HN_0003_002_000	BB22	*TP53* amplicon 19	R9	4208840
CY_SS_PC_HN_0003_003_000	BB22	*TP53* amplicon 19	R9	3611187
CY_SS_PC_HN_0003_004_000	BB22	*TP53* amplicon 19	R9	2409222
CY_SS_PC_HN_0003_005_000	BB22	*TP53* amplicon 19	R9	3987095
CY_PJET_12MU_0001_000	pJET	*TP53* amplicon 12	R9	3848342
CY_LOT1_QC_0001_000	BB25	*TP53* amplicon S0	Flongle	45573
CY_LOT1_QC_0001_001	BB25	*TP53* amplicon S0	Flongle	125446
CY_LOT1_QC_0002_000	BB25	*TP53* amplicon S0	Flongle	21844
CY_LOT1_QC_0002_001	BB25	*TP53* amplicon S0	Flongle	12242
CY_LOT1_QC_0003_000	BB25	*TP53* amplicon S0	Flongle	70747
CY_LOT1_QC_0003_001	BB25	*TP53* amplicon S0	Flongle	72964
CY_PJET_12WT_0001_000	pJET	*TP53* amplicon 12	R9	2702138
CY_PJET_RATI_0001_000	pJET	*TP53* amplicon 12	R9	2521207

* Paragon Genomics kit: https://www.paragongenomics.com/product/cleanplex-tp53-panel/

Supplementary Table 3. Sequencing information
