# Supplementary material for: Accurate detection of circulating tumor DNA using nanopore consensus sequencing
Source: NPJ Genom Med. 2021 Dec 9;6:106. doi: 10.1038/s41525-021-00272-y (PMC8660781; doi:10.1038/s41525-021-00272-y)
Supplement: Supplementary file 2 — Reporting Summary [file 41525_2021_272_MOESM2_ESM.pdf]

nature portfolio

Corresponding author(s): Wigard P. Kloosterman and Jeroen de Ridder

Last updated by author(s): Sep 24, 2021

Reporting Summary

Nature Portfolio wishes to improve the reproducibility of the work that we publish. This form provides structure for consistency and transparency in reporting. For further information on Nature Portfolio policies, see our Editorial Policies and the Editorial Policy Checklist.

Statistics

For all statistical analyses, confirm that the following items are present in the figure legend, table legend, main text, or Methods section.

n/a	Confirmed	
☐	☒	The exact sample size (n) for each experimental group/condition, given as a discrete number and unit of measurement
☐	☒	A statement on whether measurements were taken from distinct samples or whether the same sample was measured repeatedly
☒	☐	The statistical test(s) used AND whether they are one- or two-sided<br>*Only common tests should be described solely by name; describe more complex techniques in the Methods section.*
☒	☐	A description of all covariates tested
☒	☐	A description of any assumptions or corrections, such as tests of normality and adjustment for multiple comparisons
☐	☒	A full description of the statistical parameters including central tendency (e.g. means) or other basic estimates (e.g. regression coefficient) AND variation (e.g. standard deviation) or associated estimates of uncertainty (e.g. confidence intervals)
☒	☐	For null hypothesis testing, the test statistic (e.g. F, t, r) with confidence intervals, effect sizes, degrees of freedom and P value noted<br>*Give P values as exact values whenever suitable.*
☒	☐	For Bayesian analysis, information on the choice of priors and Markov chain Monte Carlo settings
☒	☐	For hierarchical and complex designs, identification of the appropriate level for tests and full reporting of outcomes
☒	☐	Estimates of effect sizes (e.g. Cohen's d, Pearson's r), indicating how they were calculated

Our web collection on statistics for biologists contains articles on many of the points above.

Software and code

Policy information about availability of computer code

Data collection	MinKNOW 3.1.20 - 3.6.5<br>Guppy 3.0.3 - 3.2.10
Data analysis	CyclomicsSeq scripts and all of its requirements for processing raw data to consensus called data are available under MIT license through Github (https://github.com/UMCUGenetics/Cyclomics_consensus_pipeline; https://doi.org/10.5281/zenodo.4709688). R and Python data analysis scripts and all of its requirements for processing and plotting the data for the current study are available under MIT license through Github (https://github.com/UMCUGenetics/CyclomicsManuscript; https://doi.org/10.5281/zenodo.4644144).

For manuscripts utilizing custom algorithms or software that are central to the research but not yet described in published literature, software must be made available to editors and reviewers. We strongly encourage code deposition in a community repository (e.g. GitHub). See the Nature Portfolio guidelines for submitting code & software for further information.

Data

Policy information about availability of data

All manuscripts must include a data availability statement. This statement should provide the following information, where applicable:

- Accession codes, unique identifiers, or web links for publicly available datasets
- A description of any restrictions on data availability
- For clinical datasets or third party data, please ensure that the statement adheres to our policy

The sequencing datasets generated during the current study are available upon request at EGA (https://www.ebi.ac.uk/ega/), under accession number EGAS00001003759. The processed datasets analyzed during the current study are available at Zenodo (https://doi.org/10.5281/zenodo.3925250).

Field-specific reporting

Please select the one below that is the best fit for your research. If you are not sure, read the appropriate sections before making your selection.

☒ Life sciences ☐ Behavioural & social sciences ☐ Ecological, evolutionary & environmental sciences

For a reference copy of the document with all sections, see nature.com/documents/nr-reporting-summary-flat.pdf

Life sciences study design

All studies must disclose on these points even when the disclosure is negative.

Sample size	The sample size was not predetermined. We sequenced 32 samples, which was sufficient as the results were highly similar between runs (e.g. figure 3a). Patient inclusion was based on availability of samples.
Data exclusions	We excluded single nucleotide variant data of "exon 12" in three samples (Control B, Patient B and Control C), as we detected the haplotype of the triple mutant in these samples.
Replication	It was not possible to replicate the majority of the findings, since all blood was typically used for a single CyclomicsSeq assay. To gain insight into reproducibility, we performed an experiment, which can be found in Supplementary Figure 3. Briefly, to evaluate person-, time- and sequencing-dependent variability in CyclomicsSeq results, CyclomicsSeq was performed three times by two different operators (only one of which had experience with the protocol) on two different days using the same insert and backbone, and subsequently each CyclomicsSeq product was sequenced on two separate MinION flow cells. The results (especially the most important one: the observed ratios) were highly similar between runs. In addition to this experiment, we also compared the findings in the patients to the golden standard (ddPCR) and the majority of the results are similar between CyclomicsSeq and ddPCR.
Randomization	Randomization is not relevant to this study, since we did not allocate the participants to groups.
Blinding	Blinding is not relevant to this study, since we did not allocate the participants to groups.

Reporting for specific materials, systems and methods

We require information from authors about some types of materials, experimental systems and methods used in many studies. Here, indicate whether each material, system or method listed is relevant to your study. If you are not sure if a list item applies to your research, read the appropriate section before selecting a response.

Materials & experimental systems

n/a	Involved in the study	
☒	☐	Antibodies
☒	☐	Eukaryotic cell lines
☒	☐	Palaeontology and archaeology
☒	☐	Animals and other organisms
☐	☒	Human research participants
☐	☒	Clinical data
☒	☐	Dual use research of concern

Methods

n/a	Involved in the study	
☒	☐	ChIP-seq
☒	☐	Flow cytometry
☒	☐	MRI-based neuroimaging

Human research participants

Policy information about studies involving human research participants

Population characteristics	Patient A: Male, age 57 at inclusion, smoker (100 smoking pack years), heavy alcohol consumption (70 units per week). Tumor location: orofarynx, tumor stage: T2N3M0, radiotherapy: 35 fx of 2 Gy, total dose 70 Gy, chemotherapy: yes, type of chemotherapy: cisplatin<br>Patient B: Male, age 56 at inclusion, smoker (37 smoking pack years), moderate alcohol consumption (3 units per week). Tumor location: hypofarynx, Tumor stage: T4aN3M0, radiotherapy: 35 fx of 2 Gy, total dose 70 Gy, chemotherapy: yes, type: cisplatin and carboplatin.<br>Patient C: Female, age 82 at inclusion, smoker (34 smoking pack years), moderate alcohol consumption (7 units per week). Tumor location: oropharynx, tumor stage T2N2cM0, radiotherapy: 35 fx of 2 Gy, total dose 70 Gy, chemotherapy: no.<br>Healthy individuals: healthy employees of the UMCU.
Recruitment	As part of the conventional care the head and neck cancer patients patients will receive a MRI with diffusion sequences. At the first visit to their treating radiation oncologist patients will be informed of the PREDICT study. If interested a researcher associated with the study will explain the details of the study and will provide study information on paper. After at least 3 days the researcher will call the patients in order to receive verbal confirmation of study participation, after this confirmation the first study procedure will be planned. On the day of the procedure the researcher will collect the written informed

consent.
Healthy individuals were recruited through the mini-donor-dienst of the UMC Utrecht. Healthy UMC Utrecht employees can donate blood anonymously every few months for research purposes.

Ethics oversight

Use of the human specimens for research purposes was approved by the Medical Ethics Committee of the UMC Utrecht (16-331, 07/125 and 20/055).

Note that full information on the approval of the study protocol must also be provided in the manuscript.

Clinical data

Policy information about clinical studies

All manuscripts should comply with the ICMJE guidelines for publication of clinical research and a completed CONSORT checklist must be included with all submissions.

Clinical trial registration

NL57164.041.16

Study protocol

The full trial protocol is available on the databases of the UMC-Utrecht. Researchers outside the UMC-Utrecht can access the protocol upon request

Data collection

1.1 Handling and storage of data and documents
The research data will be handled confidentially and encoded. After the informed consent is signed, each patient receives an unique study number and 3-letter code. These codes will not be based on the patient initials and birth date. A subject identification code list will be used to link the data to the subject. The key to the code is safeguarded by the investigator. Data will be kept for a minimum of 15 years. The subjects privacy is protected by the usage of the study number, instead of personal data. The handling of personal data will comply with the Dutch Personal Data Protection Act (De Wet Bescherming Persoonsgegevens).
1.2 Monitoring and Quality Assurance
To assure the quality and validity of the research data, an independent, qualified monitor from the Imaging department will be appointed to monitor the study procedures. The monitoring will be performed according to the NFU (Nederlandse Federatie van Universitaire Medische Centra) guidelines.

Outcomes

1.1 Primary study parameter(s)
The primary endpoint is the predictive value of DW-MRI and ctDNA concentration after the start of radiotherapy based on a single measurement during treatment. Analysis of the accuracy of DW-MRI and ctDNA quantification will be done with a ROC curve based on the change in ADC, ctDNA concentration and tumor volume between measurements.
1.2 Secondary and other study parameter(s)
- ADC level, ctDNA concentration and tumor volume pretreatment
- Change ADC level, change in ctDNA concentration and gross tumor volume during therapy
- The optimal time point for MRI scanning during treatment in order to predict treatment outcome
- Determination of effect size of ctDNA quantification for future research.
- Change in ADC and ctDNA during follow-up in relation to recurring disease.

Changes in the tumor during treatment will be described by the change in tumor volume, change in ctDNA concentration and change in MRI contrast of the tumor and surrounding tissue.
